# Non-Destructive Trace Detection of Explosives Using Pushbroom Scanning Hyperspectral Imaging System

**DOI:** 10.3390/s19010097

**Published:** 2018-12-28

**Authors:** Siddharth Chaudhary, Sarawut Ninsawat, Tai Nakamura

**Affiliations:** Remote Sensing and GIS, School of Engineering and Technology, Asian Institute of Technology, Klong Luang, Pathum Thani 12120, Thailand; sarawutn@ait.ac.th (S.N.); nakamura-tai@ait.ac.th (T.N.)

**Keywords:** hyperspectral imaging, trace detection, principal component analysis, support vector machine

## Abstract

The aim of this study was to investigate the potential of the non-destructive hyperspectral imaging system (HSI) and accuracy of the model developed using Support Vector Machine (SVM) for determining trace detection of explosives. Raman spectroscopy has been used in similar studies, but no study has been published which is based on measurement of reflectance from hyperspectral sensor for trace detection of explosives. HSI used in this study has an advantage over existing techniques due to its combination of imaging system and spectroscopy, along with being contactless and non-destructive in nature. Hyperspectral images of the chemical were collected using the BaySpec hyperspectral sensor which operated in the spectral range of 400–1000 nm (144 bands). Image processing was applied on the acquired hyperspectral image to select the region of interest (ROI) and to extract the spectral reflectance of the chemicals which were stored as spectral library. Principal Component Analysis (PCA) and first derivative was applied to reduce the high dimensionality of the image and to determine the optimal wavelengths between 400 and 1000 nm. In total, 22 out of 144 wavelengths were selected by analysing the loadings of principal components (PC). SVM was used to develop the classification model. SVM model established on the whole spectrum from 400 to 1000 nm achieved an accuracy of 81.11%, whereas an accuracy of 77.17% with less computational load was achieved when SVM model was established on the optimal wavelengths selected. The results of the study demonstrate that the hyperspectral imaging system along with SVM is a promising tool for trace detection of explosives.

## 1. Introduction

The frequency of terrorist activities has increased in the last two decades leading to a global threat which is challenging humanity. Most of the terrorist attacks use a special type of bomb known as Improvised Explosive Devices (IED) in which the explosives are stored inside metal containers. The explosives are made up of chemical compounds which have a great impact even when used in small quantities. IEDs can be grouped as military, commercial and homemade on the basis of materials used for manufacturing them. Homemade explosive mixtures can be prepared by mixing inorganic energetic oxidant salts (ammonium nitrate, potassium nitrate, potassium chlorate) with fuels like petrol, diesel, charcoal etc. [1]. The oxidant salts mentioned above are easily available on the market in the form of fertilizers. Ammonium nitrate (AN) is one of the most commonly used fertilizers for agricultural purposes and a combination with this with fuel can result in ammonium nitrate fuel oil (ANFO), dynamites that are explosive compounds. Similarly, potassium nitrate is a widely used fertilizer, but is also used for manufacturing firecrackers by mixing it with blackpowder. This type of bomb is becoming widely popular as it can be manufactured using commonly available materials at home [1,2,3,4,5,6,7,8,9]. In recent years across the globe, some major terrorist attacks took place in Pakistan, Brussels, Nigeria (2016), Paris (2015) and Boston (2013) in which IEDs were widely used. IEDs are becoming ideal weapons for spreading terrorism due to its special features like ease of manufacturing at low cost and it can be designed in various sizes and forms making it difficult to be detected.

Standoff trace detection is a method in which the operator or the sensor does not have physical contact with explosives and therefore this technology provides for a better capability for different architectures of security. Some of the potential techniques to detect traces of explosive materials are laser based spectroscopic techniques which include raman spectroscopy, laser induced breakdown spectroscopy (LIBS), resonant raman spectroscopy, laser induced fluorescence (LIF), ion mobility spectrometry and hyperspectral remote sensing [10,11]. LIBS is considered as a destructive technique and therefore it is not recommended to be used on humans and vehicles. Since the laser beam used in this technique is pointed at a specific spot, it becomes more time-consuming and difficult to scan large areas in real conditions [12]. Ion mobility spectrometry (IMS) is one of the most commonly used techniques for trace detection. Although it is a successful technique, it has drawbacks like false alarm rate, matrix effects, instability in response and use of physical molecular structure for detection [13]. LIF works on the principal of decomposing molecules into characteristic fragments [14,15] but LIF can detect only nitrogen containing explosives and requires a laser source which can be tuned to different wavelengths which results in thermal degradation of samples [16]. Many scientists and engineers from industry and academia have been working on implementing standoff trace detection methods on different applications [11,17,18,19,20,21,22,23] but no technology has emerged as an optimum technique yet. Methods which are used today to scan suspicious objects on a bomb site still have the operators at risk due to their close contact with the target [1,2,3,4,5,6,7,8,9]. Therefore, a new technique is required in order to tackle the problem of detection of target objects while maintaining the safety of the operator.

**Figure 1:**
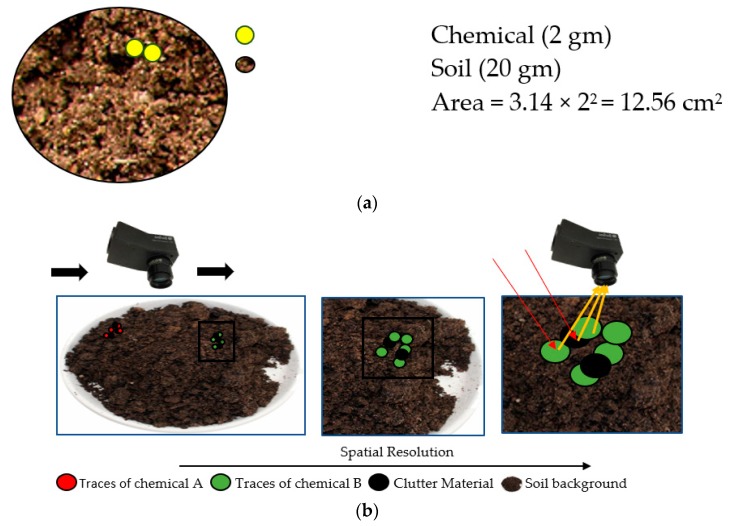
(**a**) Conceptual example of traces of chemicals present on the soil surface in a petri dish of 4 cm diameter; (**b**) Conceptual example of a standoff trace detection sensor illustrating key challenges associated with standoff trace explosive chemical detection.

Trace detection of chemicals used in explosives involves many difficulties and challenges due to the physical properties of the traces. Some of the key challenges faced by the standoff trace detection techniques are discussed below [24]:

1. Average area density (AAD)
  AAD is defined as the total mass of a chemical in a given area. AAD calculated for the traces of the chemical (2 gm) is very low as compared to the AAD of the background material (soil 20 gm). It can be visualized in conceptual Figure 1a that a small number of traces of chemicals is present on the surface of soil in a petri dish of 4 cm diameter.
  AAD for traces of chemical is calculated to be 0.15 gm/cm^2^ where as AAD for soil is 1.59 gm/cm^2^
2. The traces of chemical cover a very small area of the total area of the background. In the conceptual Figure 1b, it can be observed that traces occupy an area which is relatively very small as compared to the area of background. To overcome this problem, the sensor should have high spatial resolution (small ground sampling distance) to identify the traces, while keeping the area coverage region high.
3. Due to environmental causes, the traces of chemicals might be mixed with clutter materials which can be visualized at a microscopic scale in Figure 1b. This makes it very difficult to spatially separate the received signals from the materials. A high level of clutter materials would tend to induce noise in the spectral reflectance of the chemical traces. This problem can be resolved by designing a refined algorithm with a strong discrimination capability based on the unique spectral response of the chemicals.

Hence, identification of explosives using standoff detection is a challenging task for researchers and engineers. Therefore, the focus of defense research organizations and forensic sciences is to develop a standoff trace detection technique which overcomes the key challenges discussed above and also fulfils the following requirements [19]:

- High sensitivity and selectivity
- Potential to identify hidden explosive materials
- Minimize the risk of operators
- Non-destructive technique to avoid sample destruction
- Portable to crime scene

Hyperspectral Imaging System (HSI) has the potential to carry out the above-mentioned requirement and is a powerful emerging tool which can be used for analysis of chemical traces. The traces can be identified and visualized by analysing the difference in the spectral reflectance of the trace and its background [10]. HSI has high-speed data acquisition, a non-invasive/destructive technique which is a combination of imaging and spectroscopy. This technique has been applied in various applications such as food engineering, agriculture, airborne survey, forensic science and mineralogy [5,10,25,26,27,28]. Therefore, it is a well-established technique for object detection, monitoring the quality of the surface and studying the composition of the sample. HSI is a suitable technique for trace detection of residue of explosives due to its notable combination of imaging and spectroscopy, capability to determine the composition of each pixel generated in the cubic image within a fast response time [29] which makes it more advantageous over the current spectroscopy methods which only provide spectral information, ignoring spatial information about the traces of explosives. Individual spectra generated from HSI gives rich spectral and spatial information which works as a powerful tool for quantification and identification of the samples in a more efficient manner. The HSI system is undergoing rapid development, making the system more portable with high area coverage so that it can be used at the crime scene to view and interpret samples in real time.

**Figure 2:**
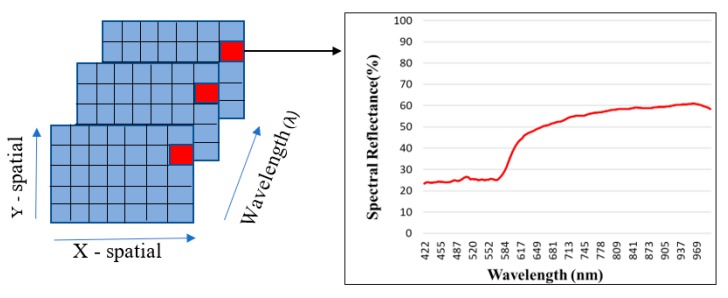
Representation of hyperspectral image as a function of wavelength and image data structure.

Images generated from the HSI technique are also known as hypercube due to their three dimensions in which X and Y represent the spatial information of the image whereas Z gives information regarding the spectral property of the sample at different wavelengths. It can also be viewed as a stack of two-dimensional images obtained at specific wavelengths. The system collects a series of image planes which maps the intensity of reflected light from the surface at a given wavelength. The hyperspectral image is composed of a large number of pixels which gives the information regarding the reflection at a wavelength and is used to generate the spectral graph as shown in Figure 2. Use of the HSI technique to perform standoff trace detection of explosives would be of great interest to defense agencies across the globe as it would solve the problem of detection of target objects while maintaining safety of operators.

When chemical samples are exposed to a source of light with proper wavelength and energy, it results in an interaction of incident photons and the surface of the [30,31]. The energy which is incident on the chemical sample can undergo several phenomena like absorption, transmission, scattering, and reflection. The ratio of reflected energy to incident energy is termed as reflectance [10]. Information regarding the chemical sample can be acquired by analyzing the amount of reflected energy which is a function of wavelength [5]. Chemical composition of the samples is a factor which determines the reflectivity and the signals reflecting from the surface of the chemical is affected by the following parameters [24]:

- Optical properties of the sample
- Illumination of light source
- Sensitivity of the detector

As discussed, reflectance is function of wavelength; therefore, all chemicals used in explosives have their own unique spectral reflectance [32]. An electronic database known as a spectral library can be generated which can store reflectance of different explosive materials using their hypercube image. Since the hypercube image and spectral reflectance is unique for every explosive material, it can be used for identifying and discriminating the various chemicals used in explosives [33,34].

In most of the studies for object classification, Support Vector Machine (SVM)—a machine learning technique—is used [35,36,37,38,39]. It is an extensively used nonlinear classification technique. The algorithm tends to minimize generalization errors by finding the optimum margin (hyperplane) between the support vectors to separate the classes. SVM has an advantage over other classification algorithms due to its performance with small samples, and a nonlinear and high dimensional dataset. This technique has been used for several applications, but not implemented for finding traces of chemicals in a hyperspectral image.

**Figure 3:**
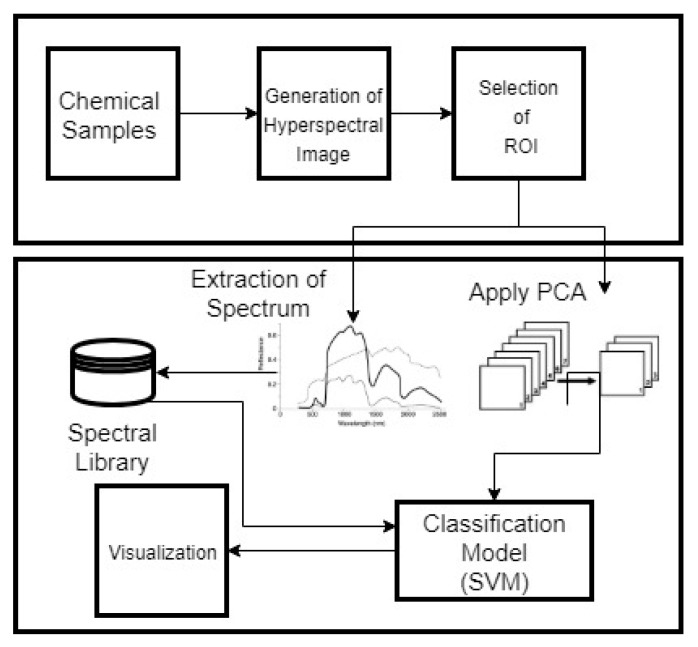
Flow of methodology.

The principal aim of the study was to develop a non-destructive system for trace detection of chemicals with high accuracy using HSI along with SVM. In this study, we also evaluate the potential of HSI for trace detection of different chemicals used in explosives and to develop a spectral library for chemicals like AN, C4 and trinitrotoluene (TNT) which are used in IEDs. In experiments, a hyperspectral sensor is used to collect spectral reflectance of the chemicals and soil at 144 wavelengths. The SVM model is later evaluated based on classification accuracy. Flow of the methodology is shown in Figure 3. 

## 2. Materials and Methods

### 2.1. Samples

In this study, composition C4, which is a plastic explosive with a texture like clay, ammonium nitrate and 2,4,6-trinitrotoluene (TNT) were used. C4 is a dirty white, light brown solid and it smells like motor oil whereas TNT is a yellow solid. Composition of C4 varies according to the manufacturers but 90% is research department explosive (RDX) and the remaining 10% is a mixture of polyisobutylene, dioctyl sebacate and mineral oil [40,41]. C4 is considered resistant to physical shocks and it can explode only when the detonator inside the explosive is exposed to fire. The chemical can be molded easily into different shapes to vary the direction of explosion. A total of 20 samples of pure chemical AN (10 gm), C4 (10 gm) and TNT (10 gm) and 10 samples of soil (20 gm) mixed with AN (2 gm), C4 (5 gm) and TNT (2 gm) were used in this study. Chemical samples were prepared after weighing them accurately. The weighted chemical samples were placed in petri dishes with a black body and a diameter of 4 cm. Average area density of AN, TNT was 0.16 gm/cm^2^, whereas for C4 and soil it was calculated to be 0.39 gm/cm^2^ and 1.59 gm/cm^2^, respectively. Samples were mixed, distributed and levelled uniformly in the petri dishes to reduce surface roughness. Petri dishes were placed over a black platform to minimize any reflection from the background.

### 2.2. Hyperspectral Imaging System

#### 2.2.1. Hyperspectral Image Acquisition

**Figure 4:**
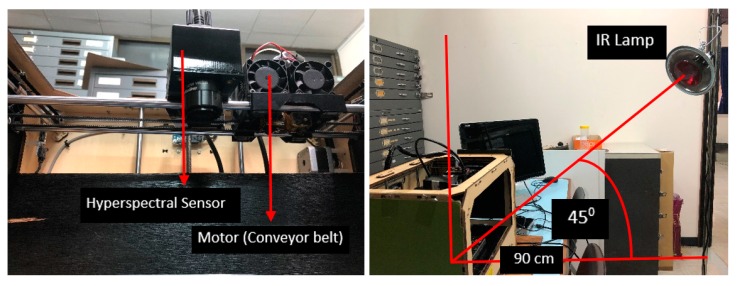
Laboratory setup of hyperspectral imaging system (HSI).

HSI was assembled at a laboratory scale to obtain hyperspectral images of the chemical samples based on reflectance. The reflectance data in the laboratory was obtained by BaySpec OCI F hypespectral sensor, which consists of a charge couple device which has a readout mode; specifications of the hyperspectral sensor are shown in Table 1. The images were acquired by pushbroom scanning in 144 bands with spectral resolution of 4.16 nm from 400 to 1000 nm using software SpecGrabber (Super Gamut, BaySpec, Inc., San Jose, CA, USA). Measurements were taken using 150 W halogen and 150 W infrared lamps as the light sources positioned 90 cm from the sample at an angle of 45° to the zenith, as shown in Figure 4. Intensity of the halogen lamp used in the experiment decreases after 700 nm, therefore the infrared lamp is used along with the halogen lamp to have uniform intensity throughout the spectrum of 400–1000 nm. For acquisition of spectral data, the sensor was placed 40 cm above the stage on which the chemical samples were placed. To complete the scanning of the chemical samples in one single scan line, the sensor was attached to a conveyor belt which moved at a speed of 1 cm/s. The hyperspectral sensor placed at the nadir detected the reflected energy in the area of 15.4 cm × 12.3 cm with a field of view of 22° at 144 bands. Spatial resolution (pixel/mm) is a function of distance between the sample and the sensor at nadir position; for the laboratory condition it was calculated to be 0.012 cm. The samples prepared were placed in black petri dishes of diameter 4 cm and placed over a black platform to minimize any sort of reflection from the background. The samples inside the petri dishes were levelled uniformly to reduce surface roughness. 

#### 2.2.2. Hyperspectral Image Calibration, Selection of Region of Interest and Extraction of Spectral Data

The white reference image was acquired using a circular white surface of teflon (8 cm diameter). This image was used for calibration of the sensor and it behaved like a perfect Lambertian surface, and in the spectral range of 400–1000 nm it achieved a minimum 95% reflection value. The dark reference image acquired by covering the camera lens with its cover and keeping all the light sources off spectral reflectance data (R) was calculated by calibrating the maximum raw intensity (I) to ideal white intensity (I_w_) and black background (I_b_).

R = (I − I_b_)/(I_w_ − I_b_) (1)

where I is intensity of the raw image, I_w_ is the intensity of the white reference image and I_b_ is the intensity of the dark reference image. CubeCreator software (Super Gamut, BaySpec, Inc.) was used to apply corrections in the hyperspectral image.

The acquired hyperspectral image is a rectangular image which needs to be cropped so that analysis is performed on the sample of interest. To perform cropping on the image, region of interest (ROI) needs to be predefined to extract the spectral information. In this study, the ROI for each chemical sample in the hyperspectral image was defined manually and spectral information for the ROI was extracted [42]. The final spectrum to represent the chemical sample was calculated by averaging the spectrum of all pixels within the ROI. This further resulted in the creation of a spectral library by putting the average spectrum of each sample together in one file. Calibration, selection of the region of interest, and the extraction of the spectral data were performed in ENVI (Environment for Visualization of Images; ITT Visual Solutions) and R studio. 

### 2.3. Data Preprocessing

#### 2.3.1. Selection of Optimal Wavelength 

HSI generates a large amount of high dimensional data which is complex and redundant, making it difficult to analyze without the support of multivariate analytical methods [43]. Principal component analysis (PCA) is a well-established statistical method [44] and an efficient technique to be applied on the hyperspectral cube to decompose the highly correlated spectral data and reduce their dimensionality. New variables called principal components (PC) are formed which are not correlated to each other and are linear combinations of the original variables [45]. Original information with minimal loss can be represented by the combination of few principal components. To reduce computational time and cost of analysis, dimensionality of the image should be reduced which can be achieved by choosing the most significant wavelengths from the spectral region where the spectral pattern differs the most [46]. PCA analysis and first derivative was applied on the hyperspectral image data to select the important wavelength. The PC generated from PCA are used to analyze the common features between the samples because samples which have similar spectral reflectance tend to cluster in the score plot of principal components [47]. Loading matrix resulting from PCA indicates the contribution of each wavelength in the PC which helps in selecting the wavelengths which are most effective in discriminating different chemical samples.

#### 2.3.2. Establishment of Supervised Classification Model

After acquisition of hyperspectral images, the reflectance values of samples of chemical and soil were imported into R dataframe and split randomly into a training and testing dataset with the ratio of 70:30, which were both used to establish the classification model. Various supervised classification techniques such as artificial neural network (ANN), random forest, and SVM are considered for classification of multispectral and hyperspectral remote sensing data. To detect traces of chemicals accurately, a classification model was built using the spectral reflectance data of chemicals. In this study, SVM was used over other classification models for identifying the traces of chemicals in the hyperspectral image because of its simple architecture, better performance with low number of inputs to the model, lower proneness to overfitting and requirement of less time and memory to store the predictive model [48,49,50,51]. Two SVM models were established in this study; the first model used all the 144 wavelengths in the spectral range of 400–1000 nm as the input, whereas the second model was based on optimal wavelengths selected. The SVM algorithm determined an optimal surface called a hyperplane which separates the classes from each other [38]. The algorithm selects the most suitable hyperplane on the basis of the maximum margin between each class. SVM is a suitable technique to work efficiently with both linear and nonlinear data with good generalization ability. For classification of spectral data, SVM had been proved as a reliable and efficient method. In this study, SVM was performed on R studio by defining the kernel functions and by determining the parameters.

The kernel function used here was radial basis function (RBF) which can make explicit the relation between the target variable and independent variable, and grid search procedure along with 10-fold cross validation was used to optimize the parameters of SVM. Classification accuracy is used to assess the performances of SVM models.

## 3. Results and Discussion

### 3.1. Spectral Reflectance Profile

**Figure 5:**
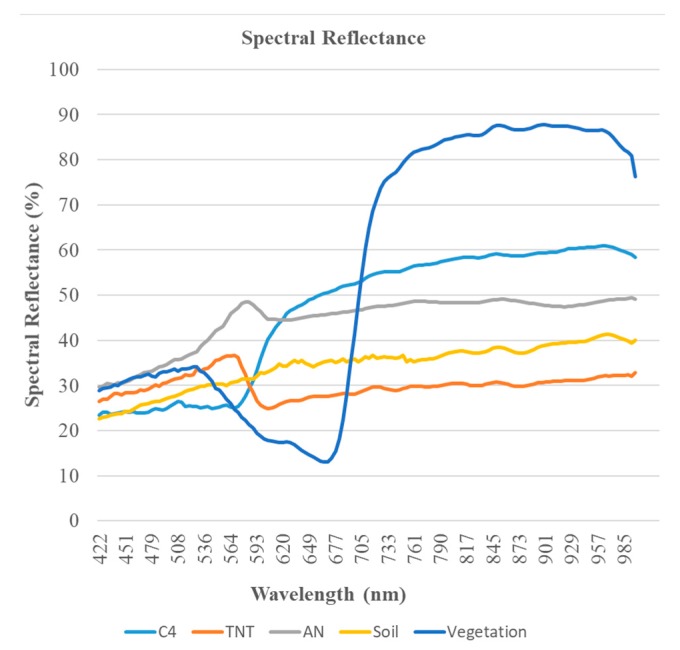
Spectral reflectance of ammonium nitrate (AN), C4 and trinitrotoluene (TNT).

Mean spectral reflectance of 20 chemical samples of AN, C4 and TNT along with soil and vegetation in the spectral range 400–1000 nm is illustrated in Figure 5. Spectral reflectance of the chemicals AN, C4 and TNT behave differently from soil and vegetation in the spectral range of 400–1000 nm which makes it easy to distinguish between explosives and non-explosives (soil and vegetation). In order to find the type of explosive used, spectral reflectance of all the three chemicals need to be analyzed. Samples of C4 and TNT showed similar trends of spectral reflectance from 400 nm till around 540 nm. From 580 nm onwards, reflectance of C4 gradually increases as compared to TNT and AN. After 572 nm there is a sharp drop in spectral reflectance of TNT, small drop for AN and it forms a valley in between 597 nm and 625 nm and remains almost constant with small change in reflectance value till 970 nm. As shown in Figure 5, chemical C4 can be differentiated from TNT and AN in the spectrum window of 580–1000 nm. Similarly, AN and TNT can be differentiated as spectral reflectance of AN is always higher than TNT. 

### 3.2. Principal Component Analysis

**Figure 6:**
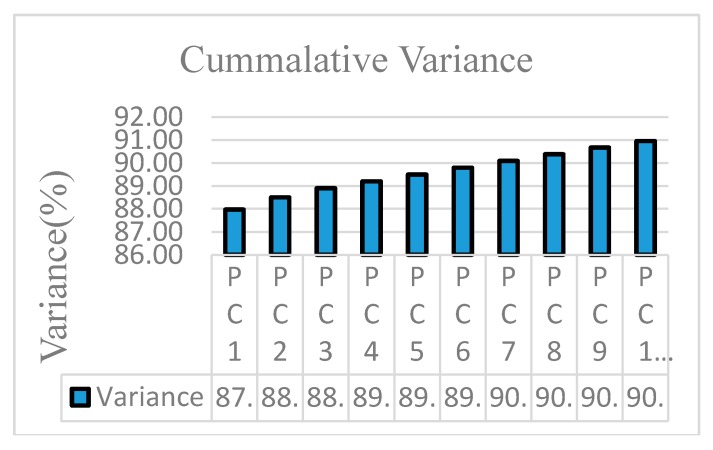
Cumulative variance of the first ten PCs.

PCA was applied on the hyperspectral image acquired by the imaging system to perform dimensionality reduction by obtaining major PCs. To construct a PCA model for the possible 60,000 spectra of each sample was computationally expensive. Therefore, to reduce the computation load, a random sample of 40,000 spectra for each sample was selected for the construction of PCA model. No significant change in the PC was observed when the number of random points were increased. PCA was performed over 144 bands and 10 PC were generated; the first four PC are listed in Table 2 for the spectrum profile between 400 and 1000 nm. PC1, PC2 and PC3 explained variance of 87.97%, 0.53% and 0.39%, respectively. The first 10 PC explained cumulative variance of 90.9% of the raw information as shown in Figure 6.

### 3.3. Optimal Wavelength Selection

The aim of optimal wavelength selection is to select only those wavelengths from a set of 144 wavelengths which carry the most important information. In this study, loading values from PCA and first derivative of spectra were used to select the optimal wavelengths.

**Figure 7:**
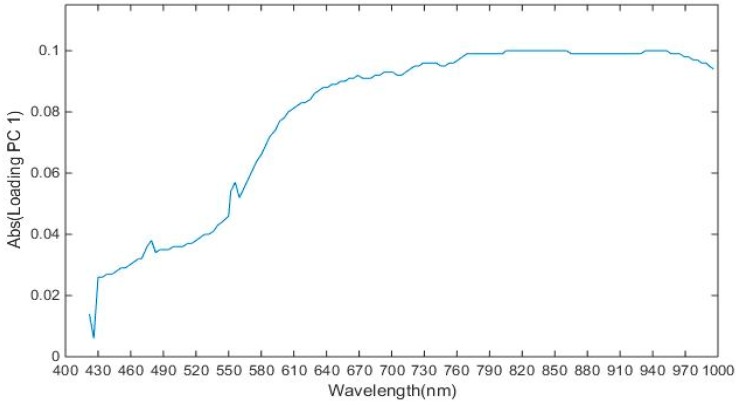
Absolute loadings for first PC.

**Figure 8:**
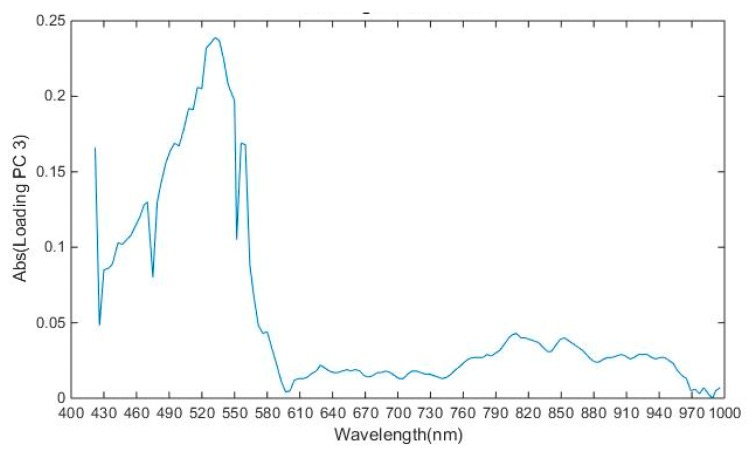
Absolute loadings for third PCs.

**Figure 9:**
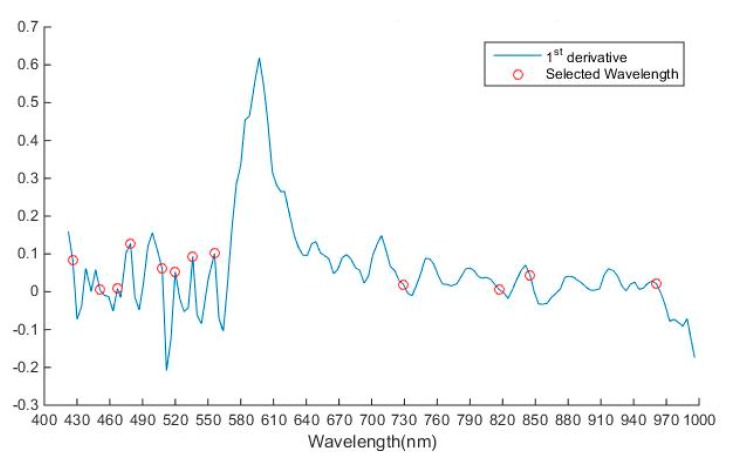
First derivative of spectra.

As discussed in the previous section, the hyperspectral image consists of large amounts of data and it also suffers from the curse of dimensionality. In this study, the dimensionality of the image was 144 and operation on all 144 bands would decrease the system performance. The spectra of the image obtained had high correlation with the neighboring bands resulting in a complex model [52]. To solve the problem of high dimensionality of the image, it was important to select the wavelengths which carry the most valuable information, so that analysis could be performed on these selected wavelengths later to improve the performance. In Table 2 and Figure 6, it was shown that variance of PC1, PC2, and PC3 explained variance of 87.97, 0.53, and 0.39, respectively and cumulative variance of first ten principal components is 90.9. To select the optimal wavelengths, loading plots of first 3 PC were analyzed. Peaks and valleys in the loading plot of PC1 and PC3 as shown in Figure 7 and Figure 8, which explain that high absolute (abs) loading values, were selected as optimal wavelengths for identifying the chemicals effectively [53,54]. The first derivative method processed the spectral reflectance of the chemical to find the wavelengths where the value of first derivative becomes zero, and these wavelengths were selected as optimal wavelengths as shown in Figure 9 [55]. Also, 22 optimal wavelengths were selected in the spectral range of 400–1000 nm using the first three PC and first derivative and are listed in the Table 3.

### 3.4. SVM Classification Model

**Figure 10:**
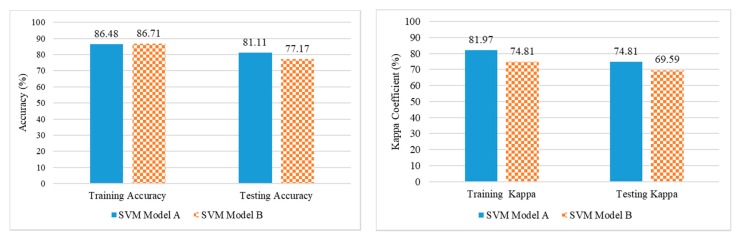
Accuracy assessment of Support Vector Machine (SVM) model on full spectra (SVM Model A) and SVM model on optimal wavelength (SVM Model B).

The spectral library generated from hyperspectral image data was used to develop a SVM model that can be used for identifying traces of chemicals and type of chemical present more accurately. The SVM model was built using caret package in R studio on the training dataset and tested on the testing dataset to check the accuracy. For developing the SVM model A, the spectral sample dataset was split into training and testing dataset with the ratio of 70:30. After splitting, 4200 spectral observations were made in the training dataset whereas 1800 observations were made in the testing data. In the spectral range of 400–1000 nm, the SVM model A achieved a discrimination accuracy and kappa coefficient of 86.48%, 81.97% for the training dataset and 81.11%, 74.81% for the testing dataset. SVM model B was developed using the optimal wavelengths selected as shown in Table 3 and it achieved the discrimination accuracy, kappa coefficient of 86.71%, 74.81 in the training dataset and 77.17%, 69.59 in the testing dataset. An accuracy assessment for both SVM models are shown in Figure 10 and class wise classification results and confusion matrix for model established on 144 wavelengths (SVM model A) and on selected wavelengths (SVM model B) are shown in Table 4 and Table 5, respectively. With the pixel size of 0.012 cm, the traces of AN, C4 and TNT were identified successfully while some pixels of soil, C4 and TNT were misclassified. Misclassification might have occurred because the difference of the spectral reflectance at certain wavelengths was not significant. 

Trade-off between cost and accuracy of the SVM model using radial function was determined by varying the cost from 0.25 to 128 and it was observed that the accuracy increased from 0.72 to 0.864, respectively. Tuning parameter sigma was held constant at a value of 4.86. Accuracy was used to select the optimal model using the largest value. The final values used for the model were sigma = 0.006 and C = 4.

Classification results of the SVM model using full spectra and optimal wavelength for identifying AN, C4, TNT and soil were satisfactory as all the four classes achieved an accuracy of more than 76% in the training and 70% in the testing dataset. Conversely, execution time of SVM model B based on optimal wavelengths was reduced to almost half than the model A based on full spectra. For all individual classes: C4, AN, TNT and soil, class accuracy was calculated to measure the performance of the classification based on the confusion matrix. There were 241, 173, 150, and 4 pixels which were misclassified during training of SVM model A gave an accuracy of 76.71%, 83.25%, 86.12%, and 99.61%. During testing of SVM model A, 131, 89, 98, and 22 pixels were misclassified, achieving an accuracy of 70.36%, 79.59%, 79.19%, and 95.12%. Similarly, individual classes in the SVM model B gave an accuracy of 76.47%, 84.32%, 86.30%, and 99.71% during training and 70.51%, 73.85%, 77.38%, and 95.90% during testing. 

## 4. Conclusions

In this study, we examined the potential of the HSI for rapid and non-destructive assessment for standoff trace detection of explosives. For the purpose of collecting hyperspectral images, halogen and infrared lamp, along with hyperspectral sensor were used. The average spectrum of different chemical samples was calculated from ROI in order to develop the spectral library of chemicals used in explosives. PCA along with first derivative method were used to select the optimal wavelengths. In total, 22 wavelengths were selected from 144 wavelengths which were used for establishing a simplified SVM model which had similar predictive accuracy as compared to SVM model developed on full spectra, but the performance of the model was enhanced because the execution time of the simplified SVM model was nearly half that of the original SVM model due to which computational load reduced. Models developed using the optimal wavelengths showed acceptable results for trace detection of chemicals with an accuracy of 77.17%, whereas the SVM model developed using spectral values from the whole spectrum of 400–1000 nm achieved an accuracy of 81.11%. Therefore, for identifying chemical traces in the large scene of ground images, we need the SVM model built using the optimal wavelengths due to its less computational load. The results of the study support the high potential of HIS along with the SVM model for trace detection of explosives by overcoming the drawbacks of existing techniques (such as being time consuming, heavy equipment, and destructive processes).

We also conclude that the result obtained in this study can be used for reference. However, we limited our scope to laboratory conditions, with an artificial light source for trace detection of three chemicals and keeping soil in the background because trace detection of explosive materials is encountered in various environmental conditions. Their analysis brings specific challenges which will be addressed in future work. In order to adapt this system for real ground conditions, a future study will be conducted under different environmental conditions and natural daylight. In particular, changing the parameters can further optimize the algorithms and improve the model for more accurate trace detection HSI in the future. 

## Figures and Tables

**Table 1:** Specifications of hyperspectral sensor.

Scanning Technique	Push Broom
**Field of View (FOV)**	22°
**Focal Length**	16 mm
**Frame Per Second (FPS)**	45 fps
**Swath**	15.4 cm × 12.3 cm
**Spatial Resolution**	0.012 cm
**Spectral Bands**	144
**Spectral Resolution**	4.16 nm (FWHM)
**Height of Sensor**	40 cm

**Table 2:** Variance of individual principal components (PCs).

Principal Component	Variance
PC1	87.97
PC2	0.53
PC3	0.39
PC4	0.30

**Table 3:** Selected optimal wavelength.

Spectral Range (nm)	Wavelengths Selected (nm) (Combining Loading and First Derivative)	Number of Wavelengths
**400–600**	426, 451, 467, 479, 508, 520, 536, 556	8
**600–800**	665, 693, 717, 729, 733, 761, 765	7
**800–1000**	805, 829, 845, 853, 865, 925, 961	7

**Table 4:** Classification results of SVM model A.

**Reference Prediction**	**SVM Model A Training**				
**Class**	**C4**	**AN**	**TNT**	**Soil**	**Accuracy (%)**
**C4**	794	150	91	0	76.71
**AN**	145	860	28	0	83.25
**TNT**	107	40	931	3	86.12
**Soil**	4	0	0	1047	99.61
	**SVM Model A Testing**				
**Class**	**C4**	**AN**	**TNT**	**Soil**	**Accuracy (%)**
**C4**	311	75	51	5	70.36
**AN**	79	347	10	0	79.59
**TNT**	56	26	373	16	79.19
**Soil**	4	2	16	429	95.12

**Table 5:** Classification results of SVM model B on optimal wavelengths.

**Reference Prediction**	**SVM Model B Training**				
**Class**	**C4**	**AN**	**TNT**	**Soil**	**Accuracy (%)**
**C4**	806	170	77	1	76.47
**AN**	135	839	21	0	84.32
**TNT**	107	41	951	3	86.30
**Soil**	2	0	1	1046	99.71
	**SVM Model B Testing**				
**Class**	**C4**	**AN**	**TNT**	**Soil**	**Accuracy (%)**
**C4**	318	80	53	0	70.51
**AN**	94	305	14	0	73.85
**TNT**	70	31	366	6	77.38
**Soil**	12	0	7	444	95.90
